# Non-suicidal self-injury in the COVID-19 pandemic: results from cross-sectional surveys among Brazilian adults from 2020 to 2023

**DOI:** 10.3389/fpsyg.2024.1357710

**Published:** 2024-07-24

**Authors:** Andre Faro, Walter Lisboa, Brenda F. Silva-Ferraz, Derek Falk

**Affiliations:** ^1^Health Psychology Laboratory (GEPPS), Department of Psychology, Federal University of Sergipe, Aracaju, Brazil; ^2^Clinical and Health Psychology Laboratory (NEPCS), Department of Psychology, Federal University of Sergipe, Aracaju, Brazil; ^3^Health Psychology Laboratory (GEPPS), Psychology Graduate Program, Federal University of Sergipe, Aracaju, Brazil; ^4^Department of Population and Quantitative Health Sciences, Case Western Reserve University School of Medicine, Cleveland, OH, United States

**Keywords:** non-suicidal self-injury, COVID-19, anxiety, depression, mental health

## Abstract

The multilevel psychosocial stressors associated with COVID-19 pandemic set the stage to investigate risk factors and groups susceptible for non-suicidal self-injury (NSSI). A national sample of 9,929 Brazilian adults aged 36.1 years on average participated in the study. Cross-sectional data were collected in 2020, 2021, 2022, and 2023. NSSI levels were considered high in the total sample (13.2%) when compared to other studies in this context. The variables with the highest explanatory power in the regression models were age, anxiety, and depression. The main risk factors were being younger, living in the South or Southeast regions of Brazil, having lower educational attainment, and having higher rates of anxiety and depression. Respondents had the highest probability of NSSI in 2022. The sustained higher rates of NSSI in 2023 compared to the beginning of the pandemic underscores the need for continuous monitoring and the development of preventive actions for self-injurious behaviors.

## 1 Introduction

COVID-19 continues to significantly impact physical and mental health worldwide despite abating in 2023 most likely due to increased access to vaccines after peaking from March 2020 to March 2022 (World Health Organization, 2023). It is estimated more than 37 million people were infected and at least 700,000 people died in Brazil from 2020 to 2023 (Brazil, 2023). The pandemic negatively impacted mental health in the general population with evidence of an increase in depressive and anxious symptomatology (World Health Organization, 2022) as well as self-injurious behaviors (Cheng et al., 2023). These findings underscore the importance of continuous surveillance of mental health outcomes during and following the pandemic to appropriately implement preventive and care measures.

The WHO estimated a global increase of at least 25% in depressive and anxious symptoms during 2020 as a result of the pandemic (World Health Organization, 2022). Multiple stressors such as isolation, limitations on work and social interactions, and reduced access to support networks, contributed to the impact on depressive and anxiety symptoms. Additionally, loneliness, fear of infection, experiences of personal and familiar suffering, grief follow bereavement, and financial worries further contributed to heightened levels of anxiety and depression. This rise of these symptoms are risk factors for self-injurious behaviors (Russell et al., 2019; Sahoo et al., 2020).

Non-suicidal self-injury (NSSI) is characterized as an intentional harm to one’s body without intention to end life. Addressing this behavior is a high priority for clinicians due to the significant distress it causes and its potential as risk factor for suicidal behavior (Lewis et al., 2022). NSSI can be understood as a dysfunctional strategy of emotion regulation (López et al., 2023) as it seems to help individuals cope with intense emotional states and disturbing thoughts that are seen as unbearable in the moment. It has been described as an attempt to escape intensive aversive thoughts by engaging in self-mutilation (Hughes et al., 2019). Highly distressing situations, such as the ongoing COVID-19 pandemic, require greater attention to the potential increase in incidence of NSSI so that care measures can be taken. Several factors can contribute to the rise in NSSI including: economic stress, social isolation, and difficulty in accessing mental health treatments (Reger et al., 2020). Interventions aimed at addressing elevated depressive and anxious symptoms, possible risk factors for NSSI resulting from the contextual factors of the COVID-19 pandemic, are crucial for vulnerable individuals and groups at risk. Females and young adults are associated with higher risk for NSSI (Hawton et al., 2021). Self-reported loneliness and social isolation also have been associated with NSSI (Russell et al., 2019). Pre-pandemic self-harm, accumulation of pandemic-related stressors, negative emotions, and lack of adaptive coping strategies are also factors associated self-harm during the pandemic (Steinhoff et al., 2021).

Periodicity was also an important factor that may be associated with an increase in NSSI due to variation in pandemic control policies related to scientific advances of COVID-19 control (Ornell et al., 2020). The initial phase of the pandemic began in 2020 with mandatory restrictions such as confinement, lockdown, and isolation, that were detrimental to mental health in the population exacerbated by insecurities and fears related to the unknown. The challenges in 2021 and 2022 were different. The initial impact of the fear of the unknown may be attenuated, or even gone, for some people but aftereffects remain. Anxiety and depression rates increased, and living with losses became exhausting and tiring (World Health Organization, 2022; Bu et al., 2023; Neves et al., 2023; Yue et al., 2023). Therefore, periodicity of the pandemic is crucial to comprehend which demands need to be addressed in order to delineate appropriate intervention strategies (Faro et al., 2020).

Although the WHO ended the pandemic emergency phase in May 2023, COVID-19 is still classified as a pandemic with virus mutations and transmission remaining under the scrutiny of researchers and health authorities worldwide (World Health Organization, 2023). The pandemic scenario still is – and probably will remain in the coming years – as a central issue to be monitored, including psychological adjustment and the impact on population mental health. In the current study, we aimed to map the risk factors for NSSI behavior in a Brazilian sample at four time points during the pandemic: 2020, 2021, 2022, and 2023. We aim to: (1) identify the sociodemographic profile related to the presence of NSSI in the sample; (2) assess the occurrence of significant anxious and depressive symptoms in this population; and (3) analyze the predictive capacity of variables related to sociodemographic profiles and mental health symptoms on the likelihood of presenting with NSSI.

## 2 Methods

### 2.1 Participants

The initial sample consisted of 10,069 participants. One hundred forty people who declared themselves to be Brazilian Asians, Indigenous, other ethnic groups, and non-binary genders were excluded from the sample due to small sample sizes. Therefore, the final sample consisted of 9,929 individuals from all 27 Brazilian states and nearly 1,400 cities.

### 2.2 Sampling strategy

The sample was composed of four surveys in Brazil during different time points (TP). TP1 data was obtained in the first week of June (2020). TP2 data was collected in the second week of March (2021), 1 year after the Brazilian government declared COVID-19 as a national public health emergency. TP3 was performed in March 2022, 2 years after the pandemic started in Brazil. Finally, the sample of TP4 was collected in March 2023, 3 years after the beginning of pandemics.

We used a convenience sampling method and used the same strategy in each of the 4 years (data collection between March and July each year; close to one-year intervals). Participants received a public invitation on social media (Instagram, Facebook, and WhatsApp) to take part in an online survey about mental health. The first screen of the survey presented an invitation and a free and informed consent form online. The average time for completing the survey was nearly 6 min in all TPs. The study was approved by the National Council for Ethics in Research [*Conselho Nacional de Ética em Pesquisa* (CONEP); 30485420.6.0000.0008].

### 2.3 Instruments

#### 2.3.1 Sociodemographic questionnaire

A questionnaire was applied to ask for characteristics of gender/sex (male, female, non-binary or other), ethnicity/self-declared skin color (White, Black, *Pardo* – Brazilian mixed ethnicity – individuals, Indigenous, Asian, and other), education level (early childhood education, high school, higher education), place of residence (city and state) and age (in years).

#### 2.3.2 Generalized anxiety disorder scale

The Generalized anxiety disorder scale (GAD-7) uses a four-point Likert scale with seven items that measure the frequency of feelings experienced by individuals over the preceding 2 weeks. Each item ranges from (0) “not at all sure” to (3) “nearly every day.” The total score is the sum of the items. Higher scores indicate greater severity of anxious symptoms. The instrument presented adequate Cronbach’s alpha coefficient (*α* = 0.916) in validation study to Brazilian Portuguese speakers (Moreno et al., 2016). In the current study, the instrument showed high indices of reliability, with Cronbach’s Alpha (α) and McDonald’s Omega (Ω) both at 0.90. Based on the total score obtained, individuals can be classified into different categories of severity: no anxiety (0–4), mild anxiety (5–9), moderate (10–14) and severe (15 or more) (Spitzer et al., 2006).

#### 2.3.3 Patient health questionnaire-9

The Patient health questionnaire-9 (PHQ-9) consists of a four-point Likert scale that measures the frequency of certain situations experienced over past 2 weeks (Kroenke et al., 2001). Each item is rated from (0) “not at all” to (3) “nearly every day.” The total score is obtained by summing up the scores of all items. Higher scores indicate a higher severity of depression symptoms. The Brazilian version obtained satisfactory reliability (α > 0.85) (De Lima Osório et al., 2009). In the current study, the instrument demonstrated strong reliability (*α* = 0.90; Ω = 0.90). From its total score, individuals can be classified as minimal (0–4), mild (5–9), moderate (10–14), moderate severely (15–19) and severe (20–27).

#### 2.3.4 Non-suicidal self-injury

A question was included in the study adapted from Self-Harm Behavior Questionnaire (Gutierrez et al., 2001). Participants were asked, “Throughout the period of quarantine and social isolation due to the COVID-19 pandemic, have you injured yourself (e.g., cutting, perforating, burning, biting, hitting) to the point of hurting yourself to relieve feelings or thoughts?” Participants responded to this question using a five-point Likert scale: (0) never, (1) rarely, (2) sometimes, (3) often and (4) many times.

### 2.4 Data analysis

Data was analyzed through JAMOVI 2.3.21 (The jamovi project, 2023). Descriptive (absolute and relative frequencies, means, and standard deviations), and inferential statistics (a binomial logistic regression, hierarchically entered) were performed. Due to the proportionally low values when considering the strata of the self-harm variable, it was decided to use it dichotomously as an outcome in the analysis (“no” versus “yes”). The dependent variable was NSSI through two outcomes: absence (never) or presence (rarely, sometimes, often, many times). The independent variables (managed as factors) were the sociodemographic features and the psychiatric outcomes: gender, ethnicity, education level, place of residence, age, anxiety, and depression. Educational level was dichotomized in up to high school and higher education (with and without postgraduate courses). The place of residence was categorized according to the official five regions of Brazil: North, Northeast, South, Southeast, and Midwest. The age was converted into five strata (quintiles): 18–24, 25–34, 35–44, 45–54 years, 55 or more. Anxiety and depression were measured according to the original cut-offs of the PHQ-9 and GAD-7. In this study, the first two strata of depression (no depression and minimal) were combined into one, following CDC standards for subclinical groups (below 10 points) (He et al., 2021). Thus, each of the psychiatric outcomes was divided into four strata: anxiety (no anxiety, mild, moderate, and severe) and depression (subclinical, moderate, moderately severe, and severe).

The final regression model was developed through an eight-block process, with one factor added in each block to assess the explanatory power of each variable individually. For the evaluation of binomial models, the fit indicators were the Omnibus test (expected to be statistically significant, indicating that the model is a good fit), the Pseudo Nagelkerke’s *R*^2^, which is expected to be as high as possible, corresponding the explained variance of the final model (from zero to one). The model was also evaluated considering AIC (Akaike Information Criterion), a comparative fit measure, in which the model with the lowest AIC value is considered the best model (Tabachnick and Fidell, 2019). Finally, the accuracy of the predict measures in final model was assessed, and higher values indicate a better fit. The reference levels values are chosen to keep all Odds Ratio (OR) above 1 to standardize the description of the findings. The level of significance was set as *p* < 0.05 for all steps.

## 3 Results

### 3.1 Sample profile

The final sample comprised 88.2% of females (*n* = 8,756) and mean age of 36.1 years (Standard Deviation [*SD*] = 13.7). More than half declared themselves to be White individuals (55.2%; *n* = 5,481). Most of the sample (81.6%; *n* = 8,098) reported having complete or incomplete higher education and the majority reported living in Northeast region (39.3%; *n* = 3,902) (Table 1). The majority consisted of individuals between 18 and 24 years old (27.1%; *n* = 2,690) the lowest stratum was composed of individuals aged 55 or older (13.3%; *n* = 1,322). The four-year groups were distributed as follows: TP1, 48.3% (*n* = 4,793); TP2, 14.9% (*n* = 1,476; 14.9%); TP3, 14.7% (*n* = 1,467), and TP4, 22.1% (*n* = 2,193). The average score of the PHQ-9 was 13.3 (*SD* = 7.51). Most of participants presented subclinical levels (35.9%; *n* = 3,562). The mean of GAD-7 scores was 11.5 (*SD* = 5.8). The biggest share of respondents demonstrated severe anxiety (35.3%; *n* = 3,502), and only 12.7% were classified as having no anxiety (*n* = 1,264). In the total sample, 1.4% (*n* = 142) reported self-harming many times during the pandemic, and 3.34% (*n* = 333) did so frequently. Additionally, 2.7% (*n* = 273) stated that they self-harmed a few times, and 5.6% (*n* = 560) did so rarely. When these responses were grouped into dichotomous terms, 13.2% (*n* = 1,308) of individuals reported exhibiting some form of self-harming behavior during the pandemic, while 86.8% (*n* = 8,621) denied engaging in such behavior. The year-by-year analysis indicated a higher prevalence of NSSI in TP3 (18.3%; *n* = 269), followed by TP2 (12.6%; *n* = 186), TP4 (12.5%; *n* = 274) and TP1 (12.1%; *n* = 579).

**Table 1:** Sample characteristics, Brazil, 2020–2023.

**Variables**		**Total sample**	**No NSSI**	**Yes NSSI**
		f% (*n* = 9,929)	f% (*n* = 8,621)	f% (*n* = 1,308)
Gender	Female	88.2 (8756)	86.5(7575)	13.5 (1181)
	Male	11.8 (1173)	89.2 (1046)	10.8 (127)
Skin color	White	55.2 (5481)	86.4 (4737)	13.6 (744)
	Pardo (mixed ethnicity)	35 (3473)	87.7 (3046)	12.3 (427)
	Black	9.8 (975)	85.9 (838)	14.4 (137)
Education level	Higher Education	81.6 (8098)	87.8 (7112)	12.2 (986)
	High School	18.4 (1831)	82.4 (1509)	17.6 (322)
Region	Northeast	39.3 (3902)	88.8 (3466)	11.2 (436)
	Southeast	38.6 (3834)	86.0 (3298)	14.0 (536)
	South	13.8 (1368)	83.6 (1143)	16.4 (225)
	Midwest	5.2 (519)	88.1 (457)	11.9 (62)
	North	3.1 (306)	84.0 (257)	16.0 (49)
Age group (years old)	18–24	27.1 (2690)	74.8 (2013)	25.2 (677)
	25–34	24.6 (2446)	87.3 (2135)	12.7 (311)
	35–44	20.5 (2032)	91.3 (1856)	8.7 (176)
	45–54	14.5 (1439)	94.0 (1352)	6.0 (87)
	55 or more	13.3 (1322)	95.7 (1265)	4.3 (57)
Year	2020 (TP1)^a^	48.3 (4793)	87.9 (4214)	12.1 (579)
	2021 (TP2)	14.9 (1476)	87.4 (1290)	12.6 (186)
	2022 (TP3)	14.7 (1467)	81.7 (1198)	18.3 (269)
	2023 (TP4)	22.1 (2193)	87.5 (1919)	12.5 (274)
Anxiety^b^	No Anxiety	12.7 (1264)	98.7 (1247)	1.3 (17)
	Mild anxiety	27.5 (2728)	93.7 (2557)	6.3 (171)
	Moderate anxiety	24.5 (2435)	86.9 (2117)	13.1 (318)
	Severe anxiety	35.3 (3502)	77.1 (2700)	22.9 (802)
Depression^c^	Minimal and mild	35.9 (3562)	96.9 (3452)	3.1 (110)
	Moderate	19.3 (1918)	91.6 (1757)	8.4 (161)
	Moderately severe	19.8 (1961)	86.1 (1689)	13.9 (272)
	Severe	25.1 (2488)	69.3 (1723)	30.7 (765)
NSSI	Yes	13.2 (1308)	-	-
	No	86.8 (8621)	-	-

^a^TP: time point. ^b^GAD-7 cut scores: no anxiety (0–4), mild anxiety (5–9), moderate (10–14) and severe (15 or more). ^c^PHQ-9 cut scores: minimal (0–4), mild (5–9), moderate (10–14), moderate severely (15–19), and severe (20–27).

### 3.2 Logistic regression models

The final logistic regression model was achieved in eight steps and obtained an explained variance of 25.9%, with satisfactory fit indices. The AIC value decrease and the Nagelkerke’s *R*^2^ value increased in all steps. The model exhibited high accuracy of predictive cases at nearly 90%. Two variables were not entered the final model: gender and ethnicity (*p* > 0.05). The variables that remained in the final model were education level, Brazilian region, age group, year, anxiety, and depression.

Individuals who only completed high school had nearly 20% higher chances of exhibiting NSSI behaviors compared to those with a higher education degree (*OR* = 1.19; *p* = 0.035). Compared to Northeast region of Brazil, individuals residing in the South region had a 36% increased likelihood in NSSI (*OR* = 1.36; *p* < 0.001), whereas those living in Southeast region had a 27% increased likelihood to it (*OR* = 1.27; *p* < 0.001). Younger individuals had higher chances of engaged in NSSI compared to those aged 55 years or more, showing a progressive increasing in the OR: from “35 to 44 years” strata (*OR* = 1.70; *p* = 0.001) to “18 to the 24 years” group (*OR* = 7.74; *p* < 0.001). People who responded in the year 2022 exhibited 2.5 times higher odds of presenting NSSI compared to those in 2020. Those who responded in 2023 (*OR* = 2.11; *p* < 0.001) and 2021 (*OR* = 1.64; *p* < 0.001) also showed elevated odds of NSSI compared to 2020. When considering explained variance in descending order, it can be observed that the age group variable has the highest value (*ΔR^2^N* = 0.098), followed by anxiety (*ΔR^2^N* = 0.079), depression (*ΔR^2^N* = 0.049), and year (*ΔR^2^N* = 0.023).

Anxiety and depression followed a similar pattern: the more severe the symptoms, the higher the likelihood of had engaged in NSSI. Individuals with mild symptomatology of anxiety had a threefold increase in the likelihood of engaging in NSSI, compared to those with no symptoms (*OR* = 3.00; *p* < 0.001). Regarding anxiety, a progressive pattern of higher odds of NSSI was observed as the symptoms became more severe: mild anxiety (*OR* = 3.00; *p* < 0.001), moderate (*OR* = 3.79; *p* < 0.001) and severe (*OR* = 4.72; *p* < 0.001). For the depression outcome, as the severity of depressive symptoms increased, individuals were more likely to engage in NSSI: moderate (*OR* = 1.71; *p* < 0.001), moderately severe (*OR* = 2.50; *p* < 0.001), and severe (*OR* = 6.28; *p* < 0.001) (Table 2).

**Table 2:** Logistic regression for non-suicidal self-injury outcomes, Brazil, 2020–2023 (*n* = 9,929).

**Steps**	*R*^2^_N_ (ΔR^2^_N_)	Variables	***p*-value**	**OR (IC 95%)**
Education level	0.009 (0.006)	Higher Education	–	–
		High School	0.035	1.19 (1.01–1.40)
Region	0.014 (0.008)	Northeast	-	–
		North	0.526	1.20 (0.77–1.86)
		Southeast	0.003	1.07 (0.78–1.46)
		South	0.004	1.36 (1.00–1.85)
		Midwest		
Age group (years old)	0.112 (0.098)	55 or more	–	–
		18–24	<0.001	7.74 (5.69–10.52)
		25–34	<0.001	3.20 (2.43–4.36)
		35–44	0.001	1.70 (1.23–2.34)
		45–54	0.379	1.17 (0.82–1.67)
Year	0.135 (0.023)	2020 (TP1)^a^	–	–
		2021 (TP2)	<0.001	1.65 (1.34–2.01)
		2022 (TP3)	<0.001	2.54 (2.10–3.06)
		2023 (TP4)	<0.001	2.12 (1.75–2.55)
Anxiety^b^	0.214 (0.079)	No anxiety	–	–
		Mild	<0.001	3.00 (1.78–5.04)
		Moderate	<0.001	3.79 (2.24–6.41)
		Severe	<0.001	4.73 (2.79–8.02)
Depression^c^	0.263 (0.049)	Minimal and mild	–	–
		Moderate	<0.001	1.71 (1.31–2.25)
		Moderately severe	<0.001	2.50 (1.92–3.26)
		Severe	<0.001	6.28 (4.85–8.14)

^a^TP, time point. ^b^GAD-7 cut scores: no anxiety (0–4), mild anxiety (5–9), moderate (10–14) and severe (15 or more). ^c^PHQ-9 cut scores: minimal (0–4), mild (5–9), moderate (10–14), moderately severe (15–19), and severe (20–27). (1) Initial AIC = 7,735; Final AIC = 6,255. ∆AIC = 1,480. Accuracy of predictive measures = 87.3%. (2) Omnibus tests: age group, year, anxiety, and depression, *p* < 0.001; region, *p* = 0.008; education level, *p* = 0.036; gender/sex and ethnicity, *p* > 0.05. 3. *R*^2^_N_ (ΔR^2^_N_). Pseudo-R of Nagelkerke for the step (Delta Pseudo-R of Nagelkerke for the specific variable).

**Figure 1:**
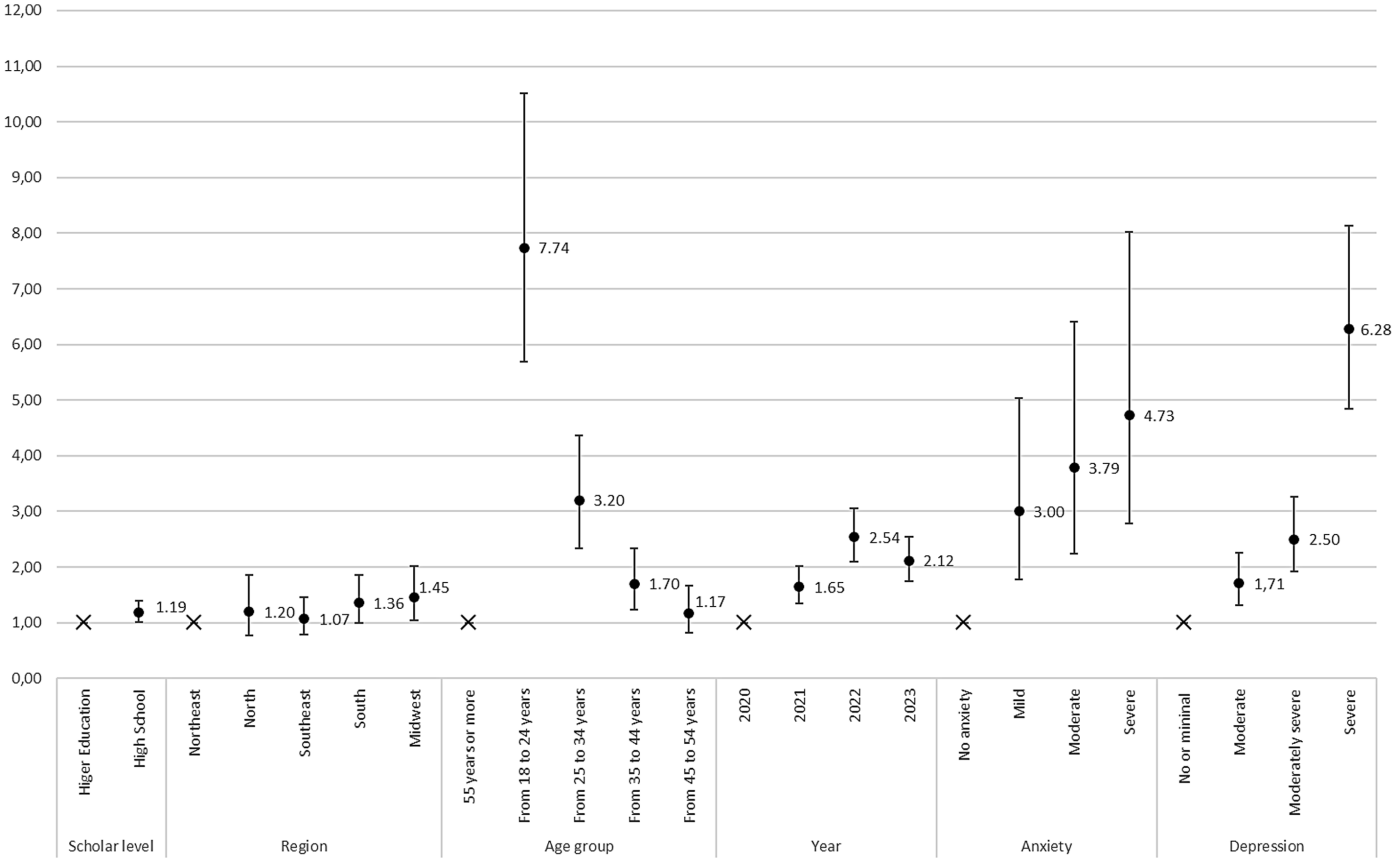
Graphic representation of logistic regression to compare odds.

Finally, to represent the most influential factors, the significant odds are graphed along two axes: variables in horizontal and odds values in vertical. Variables with highest odds were: being aged 18 to 24 years (*OR* = 7.74; *p* < 0.001), having severe depression (*OR* = 6.28; *p* < 0.001), having severe anxiety (*OR* = 4.73; *p* < 0.001), having moderate anxiety (*OR* = 3.79; *p* < 0.001) and being aged 25 to 34 years (*OR* = 3.20; *p* < 0.001) (Figure 1).

## 4 Discussion

The main aim of this study was to map the risk factors of NSSI during the COVID-19 pandemic within the Brazilian population. To achieve this aim, it was necessary to identify anxious and depressive symptoms as well as sociodemographic characteristics that indicate a higher vulnerability to the phenomenon. Additionally, the study sought to analyze the predictive capacity of these variables for NSSI.

In the total sample, 13.2% of the participants engaged in self-harm, which means that at least one in each ten people reported some level of NSSI from 2020 to 2023. At a global level, a meta-analysis of observational epidemiological studies – MOOSE (Cheng et al., 2023) found a prevalence of self-harm behavior of 15.8%, with variations among regions, namely: Asia (27.7%), Europe (10.3%), America (11.3%), and Oceania (11.2%). Compared to other countries during the pandemic, the rate of this study was lower only than Asian countries. The pandemic was a stressful situation, which may help to explains the increase in NSSI as this behavior is a strategy used to cope with difficult emotions during challenging times (Hughes et al., 2019; López et al., 2023). The emotional impact of the pandemic as well as economic uncertainty, social isolation, and difficulties in accessing mental health care, are factors that may have influenced the risk of self-harming behavior worldwide (Reger et al., 2020; Rudenstine et al., 2022; Taylor, 2022).

In 2020, 2021, and 2023, the prevalence of NSSI in this sample averaged around 12%, which is approximately six percentage points lower than in 2022 (18.3%). The pandemic cannot be considered a singular and linear phenomenon as various events unfolded over these 4 years. Each epidemiological week had its specificities, and understanding what happened during each data collection period of this study can provide a better explanation for the occurrence of self-injury. In the first weeks of June 2020, Brazil already had over 850,000 cases and more than 42,000 deaths from COVID-19 (Brazil, 2023). The country was facing an unprecedented situation of having to deal with social restriction measures to contain the virus, and the partial resumption of activities would only come in the middle of that month (Brazil, 2020). Throughout the world there has been an increase in concerns about the potential impacts of the pandemic (Boyraz et al., 2020) including living with uncertainty about the best way to prevent the spread of the coronavirus and cope with the illness (Taylor, 2022). Daily pandemic information bulletins featured real-time anxiety-inducing news (Cinelli et al., 2020) that were broadcast every night on the internet and public television. This scenario led to an increase in uncertainty (Ferreira et al., 2020), fear, anxiety, and depression (World Health Organization, 2022), prompting researchers and health organizations to refer to a pandemic of mental health issues concurrent with COVID-19 (Ornell et al., 2020).

This situation may have contributed to the prevalence of 12.1% of NSSI among participants in 2020, a statistic that increased in the subsequent years. In 2021, this rate increased to 12.6%. In January of that year, Brazil initiated a nationwide vaccination campaign (Butantan Institute, 2021) but was unable to broadly contain the spread of the virus. At the time of data collection for this research for the first time, the country surpassed a monthly accumulated death toll of 35,000 and more than 1.5 million cases of COVID-19, reaching an accumulated total of nearly 30 million cases and nearly 700,000 deaths in that month (Brazil, 2023). Several cities in the country experienced a collapse of the healthcare system, with hospitals at full capacity and people dying due to the lack of available hospital beds (Butantan Institute, 2021). At the same time, Brazil achieved a vaccination milestone with 90% of the population having received at least one vaccine dose, and 80% completing the full vaccination regimen in 2021.

In 2022, COVID-19 incidence and mortality decreased in Brazil (2023). Nevertheless, the 18% prevalence of NSSI was the highest annual value observed in this study. One hypothesis for this phenomenon is that despite the improvement in pandemic-related statistics, the country still faced alarming epidemiological figures, with an average of over 200,000 new COVID-19 cases and 1,500 deaths per week. This persistence in the incidence and mortality rates, albeit in decline, along with the cumulative stress of the pandemic could explain the increase in NSSI cases in 2022.

In 2023, the number of deaths had significantly dropped to around 300 deaths per week during data collection, a much lower number than those observed during peak weeks (Brazil, 2023). The reopening of in-person activities had expanded, and the mandatory use of masks in commercial and educational establishments was phased out gradually, with the country operating similarly to the pre-pandemic period. There was a decrease in the occurrence of NSSI observed in this year (12.5%), although it remained at a higher percentage than that observed in 2020 (12.1%). The persistence of percentages still above those observed in 2020 reinforces the need for ongoing monitoring of this phenomenon since the accumulation of stressors can be a risk factor for mental health (Louie et al., 2023) and the pandemic potentially continuing to impact mental health outcomes.

Other variables also had explanatory relevance for the self-injurious behavior phenomenon. The most powerful variable in the model was age, which accounted for approximately 10% of NSSI occurrence (ΔR^2^N = 0.098). Younger individuals had a higher likelihood of engaging in NSSI, with a progressive pattern where younger age was associated with a greater likelihood of this outcome. The association of higher anxious and depressive symptoms and younger individuals has been observed worldwide (Glowacz and Schmits, 2020; Hawes et al., 2022; Kauhanen et al., 2023). Throughout the pandemic, young adults experienced more significant mental health effects, with a higher prevalence of anxiety and depressive disorders (Glowacz and Schmits, 2020; Hawes et al., 2022). These elevated numbers can be explained by the unique characteristics of this age group, but two phenomena seem to have had a more pronounced impact in the pandemic context: uncertainty and social isolation.

Younger individuals tend to have a more active social life, which was affected in the early stages of the pandemic due to social restriction policies and the closure of non-essential activities such as bars and nightclubs. Uncertainty, on the other hand, has been present since the beginning of the pandemic (Ferreira et al., 2020), given the reduced job opportunities (Costa, 2020) and other economic hardships (Varma et al., 2021). This is exacerbated considering the life stage when individuals face increased academic and professional pressure (Glowacz and Schmits, 2020; Steinhoff et al., 2021), while having fewer coping resources (Varma et al., 2021; Brazil, 2021b), and access to mental health services and professionals (Hasking et al., 2021).

The South and Southeast regions of Brazil stood out with the highest incidence of NSSI. Pre-pandemic evidence already indicated a higher prevalence in self-injurious behavior in these regions (Aragão and Mascarenhas, 2022), and this situation may have worsened due to the impacts of the pandemic. These two regions led in the numbers of weekly-accumulated cases and deaths from COVID-19 (Brazil, 2023). These regions have a high population density, which favors greater exposure to virus transmission and increased effects of social restrictions (Rader et al., 2020). These conditions may help explain the greater impact on mental health (Simon et al., 2020). Additionally, in an Epidemiological Bulletin provided by the Ministry of Health (Brazil, 2021b), the South region had the highest suicide mortality rate in the last 10 years. Since suicide has NSSI as an important risk factor, it is essential to monitor this phenomenon, especially in those regions.

Despite its low explained variance (ΔR^2^N = 0.006) education level was also significant in the model, corroborating the literature describing lower education level’s association with greater vulnerability to illness (Raghupathi and Raghupathi, 2020; Lunde et al., 2021; Brazil, 2021b). Possible explanations are related to lower financial stability and greater social vulnerability associated with educational attainment (Alegría et al., 2018), which can be linked to less access to resources and treatments for COVID-19-related health and mental health problems. Other studies corroborate the idea that educational level and school-related factors are associated with health and may be a risk factor for NSSI. At a general level, a higher level of education can reduce mortality in adults, highlighting the importance of seeking ways to reduce health inequities (IHME-CHAIN Collaborators, 2024). Regarding the educational environment, students who receive support from teachers and who are exposed to a positive peer climate are less likely to engage in NSSI (Madjar et al., 2017). Other educational factors are the pressure/stress related to learning and the fact that older students (at senior high school) were more likely to engage in NSSI (Yang et al., 2022).

Psychiatric outcomes were among the variables that best explained the model: anxiety with approximately 8% (ΔR^2^N = 0.079) and depression with approximately 5% (ΔR^2^N = 0.049). The more severe the mental health symptoms, the higher odds of NSSI. It is important to note that anxiety was a more powerful predictor than depression, whereas severe depression had a greater impact on the chances of self-injury (*OR* = 6.28; *p* < 0.001) than severe anxiety (*OR* = 4.73; *p* < 0.001). This relationship, in which anxiety has greater explanatory power for NSSI in the sample while depression had higher odds of NSSI, suggests that during the pandemic anxiety was a more collective phenomenon while depression was more individual.

Anxiety has been described as an important risk factor for self-injury during the COVID-19 pandemic (Faro et al., 2022; Cheng et al., 2023). The pandemic was marked by the intense and rapid circulation of both true and false news in various media and on social networks from both reliable and unreliable sources (Cinelli et al., 2020). This scenario led to the emergence of the term “infodemic,” which is an excess of information to the point where it is difficult to find reliable sources of knowledge and guidance when needed (Pan American Health Organization, 2020). The pandemic gave rise to various fears and concerns about getting infected or infecting someone close, financial and social worries, fear of losing a loved one, among others (Boyraz et al., 2020) leading to the term “pandemic of fear” (Ornell et al., 2020). This scenario of fear and uncertainty, along with doubts about virus transmission and COVID-19 progression (Taylor, 2022), can help explain the widespread presence of anxiety during the pandemic and its impact on self-injury occurrence.

Depression, on the other hand, is a well-recognized risk factor for NSSI (Kiekens et al., 2019; Brazil, 2021b). This predictive role of depression has also been highlighted during the pandemic period in various studies (Park et al., 2022; Schleicher et al., 2023). In individuals with depressive symptoms, the pandemic may have heightened symptoms of hopelessness and loss of control in the population (Beck and Alford, 2014). The pandemic affected typical predictors of depression, such as chronic stressors or loss of situational control (Steinhoff et al., 2021; Hyland et al., 2022). Traumatic events such as the loss of a loved one, financial crises, disasters, or health crises typically precede depression (Stuart, 2004). As a traumatic experience disrupts people’s routines, with personal, family, and community losses, the emergence of depressive symptoms is expected (Makwana, 2019). In the face of intense and unbearable emotional states, NSSI can play a role in self-regulation to cope with intense emotional states (Hughes et al., 2019; López et al., 2023), such as those experienced during the pandemic.

## 5 Limitations

Due to the social restrictions resulting from the pandemic and the desire to reach as many cities as possible in the country, online and non-probabilistic data collection was the only feasible method at the beginning of the pandemic. Still, this data collection strategy allowed for a sample of around ten thousand people, which is a significant number in the context of large surveys of the Brazilian population. Moreover, the sample was predominantly composed of adults. Therefore, the numbers of NSSI may be even higher, as the most vulnerable groups are individuals with lower education levels, especially children and adolescents. This may have occurred for various reasons. The greater adherence of the female and young audiences to online surveys (Becker, 2017), restrictions on access to smartphones or laptops that would allow participation, or the inequality in internet access in Brazil, especially the most socioeconomically vulnerable, are important examples. Another limitation is that we did not evaluate other variables potentially relevant in the context of NSSI, such as traumatic events, feelings of defeat or entrapment, emotional dysregulation, social exclusion (Díaz-Oliván et al., 2021), dissociation (Aoki et al., 2024), and impulsivity (O’Connor and Kirtley, 2018), which can be estimated in future studies on self-injury research in Brazil. This study did not use a longitudinal design but rather a cross-sectional study with data collected at four specific time points among different samples. Thus, it was appropriate to consider the years as independent variables, and the data were analyzed considering the temporal cut-offs and not as a continuum with cause-and-effect relationships.

## 6 Conclusion

Given the persistence of stressors, anxious, depressive symptoms, and NSSI, the effects of the pandemic are still being felt and require continuous monitoring in the coming years. This surveillance is crucial as there is still concern about the post-pandemic period and cumulative issues in terms of global mental health. This scenario is not unique to the Brazilian population. It remains the responsibility of health authorities to implement mental health promotion programs aimed at the younger population and who is more vulnerable to NSSI and suffer from higher rates of anxiety or depressive symptomatology.

## Data availability statement

The raw data supporting the conclusions of this article will be made available by the authors, without undue reservation.

## Ethics statement

The studies involving humans were approved by the National Council for Ethics in Research [Conselho Nacional de Ética em Pesquisa (CONEP)]. The studies were conducted in accordance with the local legislation and institutional requirements. The participants provided their written informed consent to participate in this study.

## Author contributions

AF: Conceptualization, Data curation, Formal analysis, Investigation, Methodology, Project administration, Resources, Software, Supervision, Validation, Visualization, Writing – original draft, Writing – review & editing. WL: Investigation, Methodology, Resources, Validation, Writing – original draft, Writing – review & editing. BS-F: Conceptualization, Formal analysis, Investigation, Methodology, Resources, Writing – original draft, Writing – review & editing. DF: Funding acquisition, Methodology, Resources, Writing – original draft, Writing – review & editing.
